# Clinical Manifestations and Diagnosis of Nonalcoholic Fatty Liver Disease

**Published:** 2017-04-01

**Authors:** Mahmoodreza Khoonsari, Mohammadreza Mohammad Hosseini Azar, Ramak Ghavam, Khadijeh Hatami, Mosa Asobar, Ali Gholami, Abdolhalim Rajabi, Fahimeh Safarnezhad Tameshkel, Bahare Amirkalali, Masoudreza Sohrabi

**Affiliations:** 1 *Gastrointestinal and Liver Disease Research Center, Iran University of Medical Sciences, Tehran, Iran*; 2 *Faculty of Iranian Traditional Medical, Tehran University of Medical Sciences*; 3 *Dept. of Public Health, School of Public Health, Neyshabur University of Medical Sciences, Neyshabur, Iran*; 4 *Dept. of Epidemiology ,School of Public health ,Iran university of medical sciences ,Tehran, Iran*; 5 *Zabol University of Medical Sciences, Zabol, Iran*

**Keywords:** NAFLD, Clinical Manifestations, Sleep, Anxiety

## Abstract

**Background & Objective:**

Nonalcoholic fatty liver diseases (NAFLD) is the major cause of hepatocellular carcinoma and increases the risk of mortality. Understanding the trends of its clinical and biochemical changes is essential to identify patients with NAFLD that are at the greatest risk of nonalcoholic steatohepatitis (NASH) and cirrhosis in Iran.

**Methods:**

Patients with NAFLD confirmed by ultrasonography were enrolled into the current study. They had negative serologic markers of viral or autoimmune hepatitis, no findings in favor of metabolic liver disease, and had not received medications that affect liver, such as silymarin and Ursobil. Biochemical and clinical symptoms and histological variables were evaluated for each patient. Descriptive statistics were used to compute all variables.

**Results:**

A total of 206 patients, including 109 male and 97 female, with the mean age of 41.2 years were enrolled. The number of patients without obesity and diabetes were 34 (16.4%) and 48 (23.1%), respectively. Sleep disorder, delayed sleep, daytime sleepiness, and late dinner were noticeably common in patients with NAFLD. Furthermore, anxiety, thirst sensation, bloating, warming sensation, defecation disturbances, and upper abdominal pain were common among patients with NAFLD.

**Conclusion:**

NAFLD is a heterogeneous disorder with vast clinical presentations. It seems that anxiety and gastrointestinal problem are common among such patients. Moreover, inadvertent sleep could have a considerable effect on developing NAFLD. Patients with diabetes have more severe NAFLD, based on clinical and histological findings.

## Introduction

Non alcoholic steatohepatitis (**NASH**) is part of a broad range of common, chronic, and progressive nonalcoholic fatty liver disease (**NAFLD**). NAFLD is a clinical term indicating the deposition and accumulation of fat in liver. More than 5% of liver weight has hepatotoxic effects and could cause liver damage. The term NASH was introduced for the first time by Ludwig in 1980 for the females with obesity, without history of high intake of alcoholic beverages (1). Although NAFLD is defined as a benign condition, many studies confirmed the reverse cases (2, 3).

NAFLD is a complex metabolic condition and has been increasingly recognized as a major cause of liver related morbidity and mortality with both lifestyle and genetic risk factors. The disorder has a broad histological spectrum from simple fatty liver to a progressive hepatic injury condition, called NASH. Those who suffer of NASH are prone to a progressively advanced liver fibrosis in an almost short time (4-6). It is reported that 10%- 35% of general population have NAFLD (7). The prevalence of NAFLD and its associated risk factors may vary in different geographical regions. Population-based studies on the prevalence of NAFLD and its risk factors in the Iranian population are insufficient. The prevalence of NAFLD and NASH in Iranians varies from 2.9% to 7.1% in general population and 55.8% in patients with type 2 diabetes mellitus (8-10).

It is accepted that NAFLD is a heterogeneous disease with a variety of pathogenic pathways; therefore, It is expected that NAFLD/NASH to have different clinical manifestations. It is also mentioned in traditional medicine that liver diseases might cause different symptoms and complaints, which some of them are not related to liver disease. In fact, there is no specific symptom of NASH that reflect the advanced situation of the patients (4, 9). Therefore, the current study aimed at identifying the clinical manifestations of patients with NASH. Some noninvasive predictive scores to predict of advanced fibrosis were also introduced in the current study that was not appropriate for all patients.

## Material and Methods

**Patients**

In a cross sectional study that was conducted from March 2013 to December 2015. Cases were selected from patients referred to liver clinic of the Gastrointestinal and Liver Disease Research Center at Firoozgar Hospital, Tehran, Iran**.**

The current study included patients aged 18 and over with no history of liver surgery, bariatric surgery, and medications which affect liver such as silymarin, Ursobil, or statin. Patients with other contributory causes of liver disease such as chronic hepatitis B and C, hemochromatosis, autoimmune hepatitis, and the Wilson disease were excluded. NAFLD was defined as reporting fatty liver in ultrasonographic evaluations without other hepatopathies. 

**Clinical** **and demographic data collection**

The patients were interviewed and examined by 2 expert gastroenterologists. A questionnaire, including demographic and anthropometric data, physical examinations, and laboratory data was completed for each patient.

Weight was measured to the nearest 100 g with a scale in light clothing without shoes. Their heights were also measured by stadiometer to the nearest 0.5 cm. The body mass index (BMI) was calculated according to weight (Kg) divided by the square of height (meters). Waist circumflex (WC) was measured at midway between the uppermost border of the iliac crest and the lower border of the costal margin (rib cage). 

The quality and quantity of sleep were also asked from the patients. The quality of sleep was evaluated and scored between 0 and 10, in which <3= bad, 3 to 5= not good, 6 to 8= good, and 9to 10= very good. In this context, the falling asleep time less than 15 minutes, staying asleep without medication no obvious cause of sleep interruptions such as sleepwalking, apnea or night terrors, no feels of light sleep, fragmented or unrefreshing sleep, and sleeping during 10:00 PM to 4:00 AM were considered. The quantity of sleep was defined as the hours of sleeping during the night. The intensity of gastrointestinal symptoms, including abdominal distention and bloating, were evaluated and scored from 0 to 4, in which 0= absent, 1= mild, 2= moderate, 3= severe, and 4= intolerable.

Liver function tests including, alkaline phosphatase (ALP), serum albumin, aspartate aminotransferase (AST), bilirubin, alanine aminotransferase (ALT), platelet count, international normalized ratio (INR), total cholesterol (Chol), triglycerides (TGs), high-density lipoprotein (HDL) cholesterol, low-density lipoprotein (LDL) cholesterol, and hemoglobin A1c (HbA1c) were assayed by central laboratory of Firouzgar Hospital. The presence of diabetes mellitus (DM) and hypertension were defined based on American Diabetes Association (ADA) (11) and the Joint National Committee (JNC) criteria (12). Blood pressure was measured in 3 positions (prone, setting, and standing). Insulin resistance was assessed with homeostatic model assessment—insulin resistance (HOMA-IR) based on the following formula: [fasting glucose (mg/dL) × insulin (μU/mL)] divided by 405.

**Statistical analysis**

The data were analyzed by SPSS software for windows (IBM-SPSS, version 20.0 Chicago, IL, USA). Descriptive statistics was employed to measure all variables; data were reported as means ±standard deviation (SD) for continuous variables or frequencies, and percentages for categorical variables. 

**Ethics Approval**

This study protocol was approved by the Ethics Committee of Iran University of Medical Sciences, Tehran, Iran, with ID number: 93038626182 in accordance with the declaration of Helsinki. The procedure was explained to all the participants and a signed written informed consent forms was obtain. 

## Results

During the intervention, 206 patients were enrolled into the study. The mean±SD age of the cases was 41.2± 8.3 years, and 109 (52.7%) cases were male. The mean BMI was 31±1.5; also, 34 patients (16.4%) had not obesity and 48 (23.1 %) had diabetes mellitus. The basic characteristics of the patients are summarized in Table 1.

NAFLD, nonalcoholic fatty liver disease; FBS, fasting blood sugar; ALT, alanine aminotransferase; AST, aspartateaminotransferase; HOMA-IR, homeostatic model assessment—insulin resistance

Regarding the clinical presentations, the most common symptoms were upper abdominal pain, fatigue, thirst, and anxiety. The common clinical manifestations are illustrated in Table 2. Also, many of the patients had late onset of sleeping along with late dinner (60.2%).

**Table 1:** The Basic Characteristics of Patients With NAFLD

Variables	NAFLD Total=206
Age (year) Mean±SD	41.2 ± 8.3
Male( n)	109
BMI (kg/m2)	31 ± 1.5
Waist Circumference (cm) mean ± SD	106 ± 10
FBS (mg/dL) mean±SD	111.2 ± 30
Diabetes (%)	23.1
Hypertension (%)	26.0
Triglyceride (mg/dL) mean±SD	215 ± 28
Total cholesterol (mg/dL) mean±SD	207±30
Hypertriglyceridemia(>200 mg/dL), %	44.4
Hyper cholostremia (>200 mg/dL), %	42.5
High-density lipoprotein, (mg/dL) mean±SD	48 ± 30
Low-density lipoprotein, (mg/dL) mean±SD	70± 39
Systolic blood pressure (mmHg) mean±SD	125.2 ± 17
Diastolic blood pressure (mmHg) mean±SD	83.8 ± 14
ALT (U/L) mean±SD	65.0 ± 32
AST (U/L) mean±SD	64 ± 46
Bilirubin total (mg/dl) mean±SD	0.9 ± 0.4
Insulin level (µIU/ml) mean±SD	20.18 ± 20
Total Albumin (g/dl) mean±SD	4.2± 0.4
HOMA-IR index mean±SD	6.0±7.9

**Table 2:** Clinical Manifestations of Patients With NAFLD

Variables	NAFLD Total N (%)
Upper abdominal pain	77 (37.4)
Morning heaviness	144 (69.9)
Sleep onset after midnight	130 (63.1)
**Quality of sleep**	
Very Good	51 (24.7)
Good	90 (43.7)
Not Good	49 (23.8)
Bad	16 (7.7)
Sleep duration ≤6h	25 (12.3)
Time to falling asleep (≤10min)	66 (32.0)
**Sleepiness during day**	
No	81 (39.3)
Less than 60 min	78 (37.8)
More than 60 min	47 (22.8)
Extra ear sound	70 (34.0)
Nausea	28 (13.6)
Headache	90 (43.7)
Decrease of appetite	56 (27.2)
Thirsty	179 (86.89)
Degrees of anxiety	181 (87.8)
**Bloating**	
Absent	51 (26.2)
Mild	39 (18.9)
Moderate	64 (31.6)
Sever	41 (19.9)
Disguise	50 (24.3)
Warming sensation	130 (63.1)
Palpitation	59 (28.6 )

NAFLD, nonalcoholic fatty liver disease

## Discussion

NAFLD is defined as a condition where ≥5% to 10% of hepatocytes exhibit macroscopic steatosis by light microscopy in the absence of other etiologies of liver disease. NAFLD is one of the most common causes of chronic liver disease. The results of the current study showed how the clinical symptoms of NAFLD differ among patients. The severity of clinical and biochemical NAFLD presentations is under investigation (13, 14). Based on the previous studies, most subjects with NAFLD were asymptomatic and patients were usually diagnosed during a general checkup, when serum level of liver enzymes was found abnormal (9, 14). On the other hand, the problem of a group of patients was detected during a liver imaging assessment and, then, a consequent medical consultation revealed the symptoms that could be associated with the NAFLD (2, 7, 15). 

Regarding the symptoms, although the patients are usually nonspecific, fatigue is the common symptom. However in clinical practice, the study faced many patients with vast clinical complaints. On the other hand if human is considered as a whole; hence, manifestation of liver disease may present differently. Therefore, concerning the fact the patients were examined, which resulted in interesting clinical findings. In the present study, anxiety, thirst, feeling of changing temperature, and bloating were the most common complaints. Disguise and upper abdominal pain, particularly in the left upper quadrant, were also a common complaint. The upper abdominal pain may have sharp or dull quality. The complaint of frequent bloating, disguise, thirst, and headaches were almost present recently, before medical consultation and were usually treated as dyspepsia and reflux disease. Sleep disorder and late onset of sleeping were noticeable. 

Recently, many attempts are made to evaluate the effects of sleep quality and quantity in the development, and progression of inflammation and chronic diseases .This is almost a new issue in NAFLD, but it has a long history in the Iranian traditional medicine; as it is indicated in major reference books. Based on the Iranian traditional medicine, the best time to sleep is 10:00 PM to 4:00 AM, which is almost comparable with solar circadian. In fact, many of the patients in the current study stayed up late at night and woke up late in the morning. Brensmeier et al., reported a case–control study in which short sleep duration, delayed onset, and poor quality of sleep were common among patients with NAFLD. They also confirmed the association of NAFLD with late dinner. The association between sleep disturbances and dinner time needs further studies (16, 17). Previous studies also revealed the association between sleep apnea and NAFLD (17-19) . 

It is noteworthy that, in the current study the majority of subjects were male. Nevertheless, in many studies females were predominant; while, recent studies revealed the antecedence of male in this condition and the current results were comparable with those of them. This could be related to life style and dietary changes and the fact that males are more susceptible to the same risk factors. In addition, some studies illustrated that stress may be involved in NAFLD progress and males are prone to daily stress (17, 20, 21). Furthermore, obesity was the most frequent clinical finding and the previous studies estimated that almost 30% to 100% of the patients with NAFLD were obese. Several studies substantiated the positive correlation of WC and BMI, with NAFLD and NASH (22) (23). Obesity itself is associated with metabolic syndrome, and sleep apnea and sleep disorders (as the consequence of obesity, which cause chronic fatigue) are all among clinical presentations of NAFLD (20, 21, 24). In addition, WC is considered as an important issue in clinical setting to detect NAFLD. In a recent study, Motamed et al., declared that WC has the same power as fatty liver index (FLI) to diagnose NAFLD (25, 26) .This factor represents abdominal obesity, which in patients with NAFLD is usually, but not always, higher than normal. Hence, patients with lean body NAFLD/NASH require more attention. 

Blood pressure is considered as a manifestation of metabolic syndrome and NASH. In the current study, the mean blood pressure was about normal (125/83 mmHg). An exact cannot be offered, but it could be due to the limited number of participants. In former studies, the presence of DM along with other factors, such as higher BMI, hypertension and older age, were associated with severity of NAFLD/NASH (27). In the current study, according to Table 1, the mean age and BMI of the patients with NAFLD were 41 years and 31.4, respectively; the prevalence of DM was 23%, which were comparable with the previous reports (3). In fact, it seems that the age of NAFLD and NASH onset might be reduced, as reported by some studies (8, 28) . Several population-based studies, however, revealed the increasing trend of NAFLD and NASH with age (2, 13). 

In conclusion, NASH could be presented with different clinical manifestation as long time before the diagnosis .Although fatigue is a common complaint of patients, the consultant must consider other related symptoms. Furthermore, it seems that NASH is not actually a female prominent condition. Moreover, poor quality of sleep and sleep disorder could be considered as the risk factors that should be investigated. 
